# Drought stress memory in a germplasm of synthetic and common wheat: antioxidant system, physiological and morphological consequences

**DOI:** 10.1038/s41598-023-35642-2

**Published:** 2023-05-26

**Authors:** Azadeh Amini, Mohammad Mahdi Majidi, Niloofar Mokhtari, Mehdi Ghanavati

**Affiliations:** 1 Department of Agronomy and Plant Breeding, College of Agriculture, Isfahan University of Technology, Isfahan, 8415683111 Iran; 2 Department of Agriculture, Payame Noor University (PNU), P.O Box 19395-3697, Tehran, Iran

**Keywords:** Plant breeding, Quantitative trait, Genetics

## Abstract

Plants have evolved mechanisms of adaptation to fluctuations in their environmental conditions that have been given the term “stress memory”. Synthetic wheat offers new hope for breeders to restore useful genes lost during the genetic bottleneck. We aimed to test whether drought priming and seed priming could improve drought tolerance in a diverse germplasm of synthetic and common wheat under field conditions. In this research, 27 wheat genotypes (including 20 synthetics, 4 common local and 3 common exotic bread wheat) were field evaluated under four water environments. These treatments included: 1) normal condition (N), plants were irrigated when 40% of the total available soil water was depleted from the root-zone, 2) seed priming-secondary stress (SD2), only water stress was applied at anthesis when 90% of the total available soil water was depleted and seeds were planted for evaluating, 3) primary stress- secondary stress (D1D2), primary water stress was applied at jointing stage when 70% of the total available soil water was depleted then secondary water stress was applied at the anthesis stage when 90% of the total available soil water was depleted, and 4) secondary stress (D2) only water stress was applied at the anthesis when 90% of the total available soil water was depleted. Our results indicated that improved efficient enzymatic antioxidant system leads to less yield reduction in D1D2 treatment. However, the positive effects of drought priming were more pronounced in drought primed (D1D2) than seed primed treatment (SD2). Synthetic wheat genotypes had a significant superiority in terms of yield, yield components and drought tolerance compared to common wheat genotypes. Nevertheless, the response of genotypes to stress memory was very different. Drought sensitive genotypes had better response to stress memory. Superior genotypes were identified as high yield and drought tolerant genotypes which can be used for future studies.

## Introduction

Droughts are often regarded as major threats to ecosystems under global climate change^1^. More or less 45% of the world cultivated area is faced with frequent and continuous drought^2^. However, plants may undergo different physiological and morphological adaptations for acquiring tolerance to drought stress. Stress memory is a phenomenon evolved in plants to adapt to fluctuations in their environmental conditions^3^. The mechanisms behind this phenomenon involve complex, multi-level responses which manifest themselves in the form of altered physiological signaling and protective metabolites, as well as epigenetic modifications^3^. Ultimately, stress memory may provide a mechanism for acclimation and adaptation^4^.

Bread wheat (*Triticum aestivum* L., 2n = 6x = 42, AABBDD) is the most important cereal crop with significant increase in contribution of human food^5^. The major limiting factor for wheat improvement is the narrow genetic variation in common cultivars, especially in the D genome. To enhance the genetic variation in this genome, synthetic hexaploid wheat (SHW) was artificially created by simulating the evolution of bread wheat by crossing different durum or emmer wheat cultivars (AABB) with *Aegilops tauschii* (the wild diploid progenitor of the D genome)^6^. Genetic diversity in the *Ae. tauschii* populations is considerably higher than that in the D genome of bread wheat, with many useful genes for biotic and abiotic stresses and seed storage protein. Studies showed that synthetic derivative lines (SDLs) provide up to 46% increase in grain yield compared to their common wheat parents under water deficit conditions^7,8^. In Australia, increases of 9–31% in grain productivity, in comparison with parental genotypes and local control varieties, were achieved under rain-fed conditions in synthetic lines^9^. Similar grain yield increase was also achieved in Argentina, Ecuador, India and, Pakistan. Additional genetic variation for salt tolerance and tolerance to higher temperatures has been reported in SHW^10–12^, which is limited in cultivars of common wheat lines.

Recent reports in some crops have showed that the plants pre-exposed to environmental stress can achieve the potential to display a stronger and faster activation of their defense system in response to the subsequent stress challenges^13,14^. An early drought episode showed an improved photo-protection and higher production under a second drought event than non pre-exposed plants in *Arrhenatherum elatius*^1^. Similarly, pre-exposure of vegetative stage plants to heat stress improved the thermo-tolerance during grain filling in rice^15^. Likewise, stress memory benefits have also been reported in radish^14^, tobacco^16^, and some grass species^17^. A few studies addressing the inheritance effects of pre-stress priming in wheat. Primed seeds exhibited greater stress-tolerance and an enhanced germination ability over non-primed seeds under multiple adverse environmental conditions in wheat^18,19^. In another wheat study, plants which were exposed to early stage heat stress, displayed an improved antioxidative activity and higher seed yield under heat stress^20^. Li et al^21^ investigated that pre-treatment for water logging during vegetative growth stage enhanced dry matter accumulation and its distribution to grain formation in wheat. Positive effects of drought priming by alleviating drought stress and heat stress during the grain-filling stage has been recently reported in two cultivars of wheat^22^. Improvements in photosynthetic capacity, seed yield, oxidative stress reduction^20^, alleviation of photo-inhibition^23^ and enhancements in regulation of growth hormones^24^ at grain-filling stage are also attributed to drought priming.

Up to now, experiments investigating these stress imprint have been mostly restricted to small time spans of less than one week and on limited common wheat cultivars. To the best of our knowledge, no information is available about stress memory in synthetic hexaploid wheat. In addition, there is no study in terms of stress memory with this number of genotypes under field with the emphasis of agronomic and physiological traits. Therefore, we aimed to investigate whether the effect of seed priming and mild drought priming could be responsible for improving performance, physiological and antioxidant system under drought in different types of synthetic and commercial wheat cultivars under farm conditions.

## Results

### Analysis of variance

Analysis of variance (ANOVA) indicated that year effect was not significant for most of traits. Therefore, data of two years were pooled and ANOVA were performed based on two-way analysis (Combined analysis of treatments and genotypes). There were significant differences (P < 0.01) between treatments for most of the measured traits with the exception of spike length, plant height, catalase and relative water content. The effect of genotype was also significant for all of the functional and phenological traits and some physiological traits including proline, carbohydrate, catalase, ascorbate, peroxidase activities and relative water content indicating significant variation among the selected genotypes (Tables S2 and S4). Genotype × treatment effects were significant for the traits including days to pollination, proline, catalase, ascorbate peroxidase and peroxidase activities (Tables S2 and S4).

### Means of moisture treatments for functional and physiological traits

The results showed that secondary drought stress significantly reduced grain yield and yield components under three stress conditions (SD2, D1D2, D2) compared to normal condition (N) (Table 1). This reduction was 43% in (SD2), 35% in (D1D2) and 47% in (D2) for grain yield. The lowest yield reduction was related to the primary stress-secondary stress (D1D2) (Table 1). The main difference between this moisture treatment (D1D2) with other moisture treatments (SD2 and D2) is primary stress (D1) which was applied at joining stage. It seems that stress memory had been an important factor in preventing yield reduction. In addition, D1D2 treatment had a higher number of spikes and harvest index compared to other stress conditions (SD2, D2) and the effect of primary stress was quite evident for days to heading (Table 1).

**Table 1:** Mean comparison of functional and phenological traits in 27 of synthetic and common wheat genotypes under four moisture environments to evaluate drought stress memory

Treatment	Grain yield (g/m^2^)	Biological yield (g/m^2^)	Thousand seed weight (g)	Number of spikes ( m^2^)	Harvest index	Spike length (cm)	Plant height (cm)	Days to heading	Days to pollination
N	1848.12^a^	4819.4^a^	38.42^a^	1980.13^a^	38.43^a^	11.65^a^	109.32^a^	173.02^a^	183.65^a^
SD2	1049.72^c^	3568.9^b^	25.80^b^	1124.70^c^	29.39^c^	11.65^a^	109.40^a^	173.45^a^	180.49^b^
D_1_D_2_	1195.62^b^	3691/9^b^	27.76^b^	1281.02^b^	32.88^b^	11.26^a^	107.70^a^	170.28^b^	179.71^b^
D_2_	978.86^c^	3091^c^	21.45^c^	1048.78^c^	27. 06^c^	11.46^a^	108.14^a^	172.70^a^	178.46^b^

Normal condition (N), seed priming-secondary stress (SD2), primary stress-secondary stress (D1D2) and secondary stress (D2)**.**

Means followed by the same letter in each column are not significantly different according to LSD test (probability level of 5%).

There were two points sampling for physiological traits in this study as mentioned before. Firstly, at recovery period from normal (N) and primary stress—secondary stress (D1D2) treatments. Secondly, at end of second stress (D2) from all of treatments (N, SD2, D1D2, D2). The results of recovery sampling showed that the effect of treatments was significant for chlorophyll a, total chlorophyll (a + b), proline, carbohydrate and ascorbate peroxidase enzyme (Table S3). The level of chlorophyll a, chlorophyll (a + b), proline, carbohydrate and ascorbate peroxidase enzyme (APX) even after four weeks of the primary stress (D1) were significantly higher in (D1D2) treatment camper to normal treatment (N) (Table 2). Furthermore, the results of second sampling showed that the level of ascorbate peroxidase (APX) and peroxidase activity (POX) were significantly higher in (D1D2) treatment compare to others treatments (N, SD2, D2). The level of proline and carbohydrates in stress treatments (SD2, D1D2, D2) were significantly higher than the normal treatment (N) (Table 2).

**Table2:** Means of physiological traits of synthetic and common wheat genotypes at recovery period after primary drought stress (a) and at end of secondary drought stress (b).

Treatment	Chl-a (mg/g leaf)	Chl-b (mg/g leaf)	Chl-a + b (mg/g leaf)	Cars (mg/g leaf)	Proline (µmol/g leaf)	TSC (mg/ml)	CAT (µmol min/mg/protein)	APX (µmol min/mg/protein)	POX (µmol min/mg/protein)	RWC
**a: After primary drought stress**										
N	1.72b	0.506a	2.23b	0.566a	2.07b	1139.81b	2.26a	15.93b	23.86a	–
D1D2	1.99a	0.524a	2.51a	0.611a	4.38a	1533a	2.02a	32.64a	24.83a	–
**b: At end of secondary drought stress**										
N	1.30b	0.396ab	1.69b	0.468bc	0.89b	925.4b	3.12b	9.07c	12.41c	72.68a
SD2	1.43ab	0.450a	1.88ab	0.524ab	8.23a	1337.5a	3.56ab	10.86b	17.56ab	70.01ab
D1D2	1.07c	0.328b	1.40c	0.409c	7.55a	1389.5a	3.66a	12.24a	18.09a	70.21a
D2	1.56a	0.444a	2.00a	0.556a	6.19a	1361.7a	3.49ab	10.82b	16.27b	66.47b

chlorophyll a (Chl-a), chlorophyll b (Chl-b) and carotenoids (Cars) concentrations, total soluble carbohydrates (TSC), catalase (CAT), ascorbate peroxidase (APX), peroxidase (POX) activities, relative water content (RWC). Normal condition (N), seed priming- secondary stress (SD2), primary stress-secondary stress (D1D2) and secondary stress (D2). Mean followed by the same letter in each column are not significantly different according to LSD test (probability level of 5%).

### Means of genotypes or functional and physiological traits

**Figure 1:**
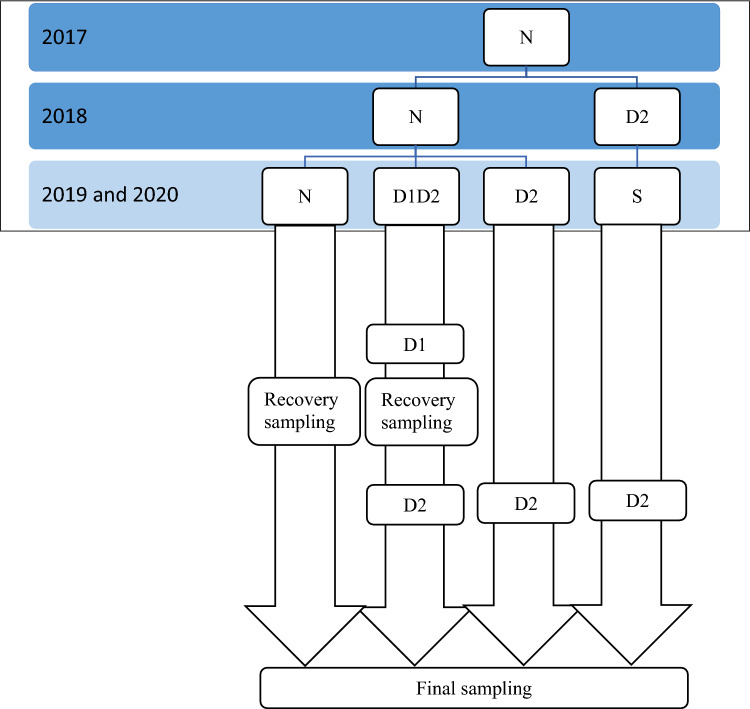
General steps and designing of this study: Seed of wheat lines were collected and reproduced during 2017. Then the lines were evaluated in the field under two treatments (normal condition (N) and intensive drought stress at anthesis stage (D2)) in 2018. Then the seed of both treatments were collected and evaluated in the field under four treatments of normal condition (N), seed priming-secondary stress (SD2), primary stress-secondary stress (D1D2) and secondary stress (D2) in 2019 and 2020.

**Figure 2:**
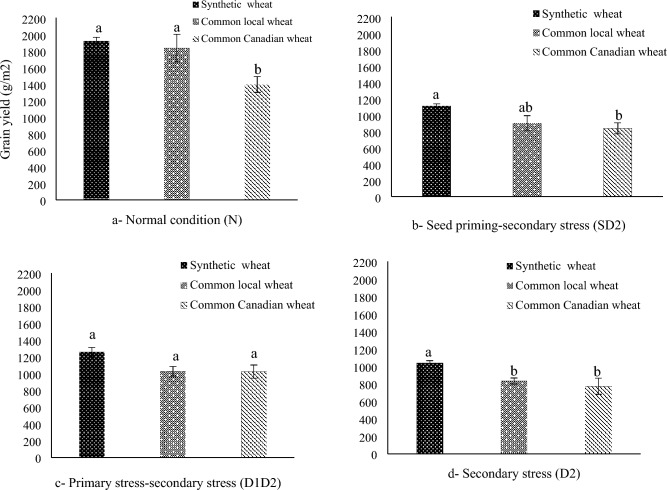
Comparison of grain yield of three groups of 27 wheat lines (synthetic, common local (Iranian) and common Canadian) under four moisture environments (Normal condition (N), Seed priming-second stress (SD2), Primary stress-secondary stress (D1D2) and secondary stress (D2)).

**Figure 3:**
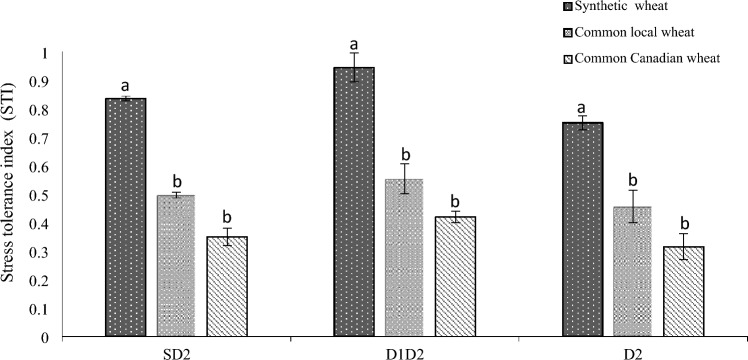
Comparison of stress tolerance index (STI) of three groups of 27 wheat lines (synthetic, common local (Iranian) and common Canadian) under four moisture environments (Normal condition (N), Seed priming-second stress (SD2), Primary stress-secondary stress (D1D2) and secondary stress (D2)).

The results showed that there were significant differences among the studied genotypes for the most measured traits (Tables S2, S3 and S4). Synthetic wheat genotypes had a significant superiority in terms of yield, yield components and drought tolerance compared to common wheat genotypes (Figs. 1, 2 and 3). While synthetic wheat genotypes had higher grain yield in normal condition (N), seed priming-second stress (SD2), and secondary stress (D2), the difference between the three genotypic groups (synthetic genotypes, common local genotypes and common Canadian genotypes) was not significant under primary stress-secondary stress (D1D2) (Fig. 2). In this respect, genotypes 200, 80, 196, 159 in normal environment and genotypes 54, 200, 7, 80 in SD2 environment and genotypes 54, 80, 196, 200 in D1D2 environment and genotypes 58, 80, 82,196 in D2 environment were identified as the superior ones in terms of grain yield (Table 3). The highest values of drought tolerance index (STI) were observed for 200, 80 as synthetic genotypes and the lowest values were detected for 4, 1 as common Canadian genotypes (Table 3).

**Table 3:** Mean comparison of grain yield and stress tolerance index (STI) in 27 genotypes of wheat.

Grain yield (g/m2)					STI		
Genotype codes	N	S	D1D2	D2	S	D1D2	D2
Synthetic wheat							
200	2428.3	1351.7	1356.7	992	0.946	0.967	0.707
80	2351.7	1280	1643.3	1229.3	0.879	1.04	0.846
196	2275	1106.7	1552.5	1205.3	0.74	0.802	0.809
159	2211.7	1056.7	1151.7	1013.3	0.677	0.749	0.651
8	2133.3	1115	1330	1117.3	0.697	0.833	0.7
58	2110	1253.3	1290	1321.3	0.813	0.802	0.792
154	2055	1046.7	1121.7	1096	0.645	0.668	0.66
7	2016.7	1336.7	1055	1109.3	0.797	0.608	0.649
199	1990	1153.3	1348.3	888	0.68	0.794	0.51
54	1981.7	1355	1695	1046	0.775	0.97	0.638
82	1890	1266.7	1141.7	1208	0.705	0.621	0.668
17	1886.7	983.3	1138.3	1049.3	0.546	0.644	0.568
173	1793.3	1105	955	1052	0.581	0.502	0.559
65	1730	850	1057	908	0.433	0.536	0.465
14	1723.3	1205	941.7	1001.3	0.603	0.487	0.518
198	1710	896.7	1275	1110.7	0.428	0.642	0.549
102	1646/7	1128.3	1341.7	964	0.554	0.645	0.467
85	1571.7	1031.7	1263.3	865	0.473	0.584	0.4
27	1518.3	765.8	1153.3	694.7	0.369	0.533	0.3
135	1363.3	946.7	1275	896.7	0.38	0.521	0.367
Local wheat							
1000	1851.7	1045	1017.5	894.7	0.606	0.568	0.527
2000	1967	1193.3	1125	928	0.687	0.648	0.534
3000	1827.5	725	936.7	685.3	0.391	0.498	0.367
4000	1685	635	1023.3	820	0.302	0.498	0.396
Canadian wheat							
4	1097.5	650	925	673.3	0.214	0.293	0.221
1	1446.7	817.3	830	912.7	0.333	0.337	0.372
2	1636.7	1041.7	1320	729.3	0.5	0.628	0.352
LSD	585.6	479.2	374.2	344.7	0.358	0.301	0.272

Normal condition (N), seed priming-secondary stress (SD2), primary stress-secondary stress (D1D2) and secondary stress (D2)**.** Mean followed by the same letter in each column are not significantly different according to LSD test (probability level of 5%).

Although three genotypic groups had specific behaviors for functional traits, there were not specific behaviors for physiological traits among three genotypic groups. In this respect, among 10 wheat genotypes (6 synthetic wheat genotypes, 2 common local and 2 common Canadian wheat genotypes) in normal treatment (N) genotypes 14, 2, 4, 58 and in seed priming-secondary stress (SD2) treatment genotypes 4, 58, 14, 2000 and in primary stress- secondary stress treatment (D1D2) genotypes 4000, 58, 2, 4 and in secondary stress treatment (D2) genotypes 159, 85, 2000, 58, 4 had the highest peroxidase enzyme activity (POX) (Tables 4). It seems that genotypes 4 and 4000 which were detected as lowest values of STI had evident enzyme activities. On the other hand, genotype 58, which was one of the genotypes with highest values of STI, had an active antioxidant system.

**Table 4:** Mean comparison of ascorbate peroxidase (APX), peroxidase (POX), catalase activities (CAT) and proline in 10 genotypes of wheat.

Genotype codes	POX (µmol/mg/protein)				APX (µmol/mg/protein)			
	N	S	D1D2	D2	N	S	D1D2	D2
Synthetic wheat								
58	14.32	23.96	25.73	17.87	10.38	11.17	12.52	9.65
198	12.78	17.37	12.07	9.97	10.96	10.16	8.97	8.19
159	12.12	10.24	13.15	22.9	6.89	10.35	14.09	14.08
196	9.93	13.83	12.88	12.92	7.93	11.93	6.62	9.94
85	8.45	12.48	7.5	21.16	6.95	9.43	9.69	11.89
14	18.15	20.12	14.11	14.21	9.36	8.23	11.55	9.97
Canadian wheat								
2	17.09	13.54	22.9	15.83	12.01	12.19	10.08	11.02
4	16.6	25.9	19.82	17.47	11.77	14.03	16.4	11.25
Local wheat								
4000	7.44	18.27	34.85	11.43	6.93	11.87	20.9	11.32
2000	7.23	19.86	17.94	18.97	7.47	9.43	11.59	10.86
LSD	1.72	2.23	2.61	1.52	2.92	2.37	2.04	3.1

Genotype codes	CAT (μmol/mg/protein)				Proline (μmol/g leaf)			
	N	S	D1D2	D2	N	S	D1D2	D2
Synthetic wheat								
58	2.87	3.54	4.24	3.8	1.01	12.46	13.25	11.56
198	4.23	3.86	3.59	3.16	0.906	5.76	3.16	3.91
159	3.41	3.02	3.72	3.5	0.969	9.33	11.5	5.04
196	3.53	4.69	3.51	3.39	0.574	2.6	7.41	2.86
85	2.75	3.15	3.33	2.47	0.686	3.75	8.11	1.76
14	3.57	3.79	3.11	3.28	0.709	7.46	8.13	5.79
Canadian wheat								
2	2.44	2.75	3.16	3.6	0.559	2.21	4.02	3.47
4	3.38	3.22	3.5	3.79	0.806	6.67	8.13	4.5
Local wheat								
4000	2.94	4.13	5.03	3.94	1.68	14.43	3.87	13.98
2000	2.1	3.48	3.42	3.94	1.02	17.67	7.74	9.04
LSD	1.41	0.7	1.22	1.26	0.513	2.59	5.48	4.45

**Figure 4:**
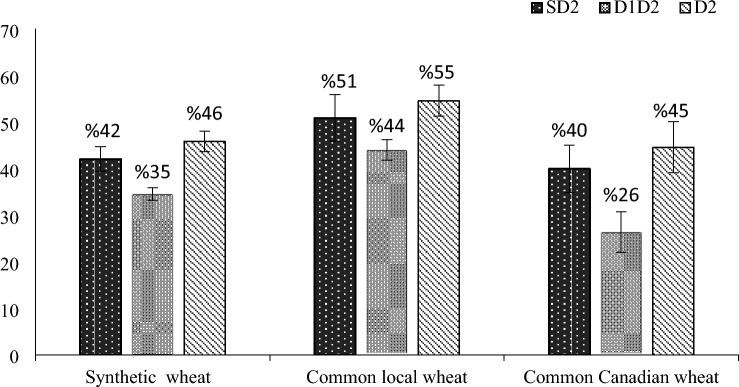
Percentage of grain yield reduction under three moisture stress environments in 27 synthetic and common wheat genotypes. SD2: Seed priming-secondary stress, D1D2: Primary stress-secondary stress and D2: Secondary stress.

The response of genotypes to stress memory (D1D2 treatment) was also very different among genotypes. In fact, yield reduction was 34% in synthetic wheat, 44% in common local wheat, 26% in common Canadian wheat (Fig. 4). Thus, Canadian wheat, which often had the lowest yield in four moisture environments (N, SD2, D1D2, D2) (Fig. 2), had the lowest yield loss in drought primed plants (D1D2) (Fig. 4). Moreover, by comparing rank of each genotype based on grain yield (Table 3), yield components (Table S5), drought tolerance index (STI) (Table 3) and physiological traits (Table 4), three synthetic genotypes (200, 80, 58) were identified as high yield and drought tolerant genotypes.

### Principal component analysis

**Figure 5:**
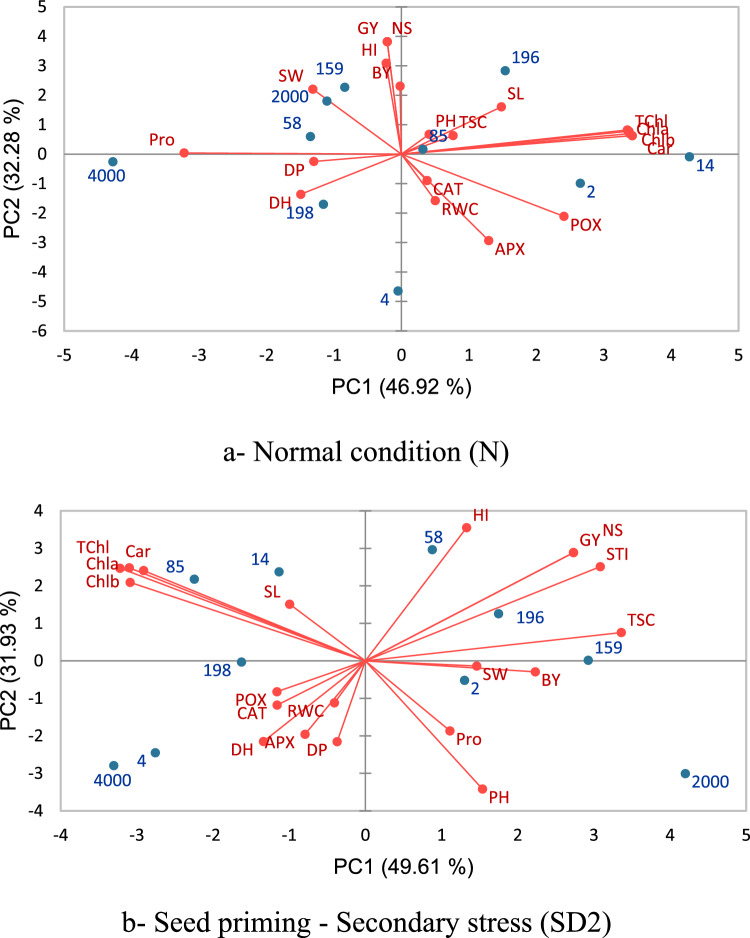
Principal component analysis of traits measured in the 10 synthetic and common wheat genotypes (**a**) under normal (N) and (**b**) seed priming- secondary stress (SD2) conditions. Horizontal and vertical axes are the first and second principal components, respectively. DH, days to heading; DP, days to pollination; PH (cm), plant height; SL (cm), spike length; NS, spike per m2; GY (g/m2), grain yield; SW (g), thousand-grain weight; BY (g/m2), biological yield; HI (%), harvest index; STI, stress tolerance index; RWC (%), relative water content; POX (µmol/mg/protein), peroxidase; APX (µmol/mg/protein), ascorbate; CAT (µmol/mg/protein), catalase; Pro (µmol/g leaf) Proline; TSC (mg/ml), total soluble carbohydrates; Chl-a (mg/g leaf), chlorophyll a; Chl-b (mg/g leaf), chlorophyll b; TChl (mg/g leaf), total chlorophyll a and b; Cars (mg/g leaf), carotenoids concentrations. The Number of genotypes is according to the first column (Genotype code) in table S1.

**Figure 6:**
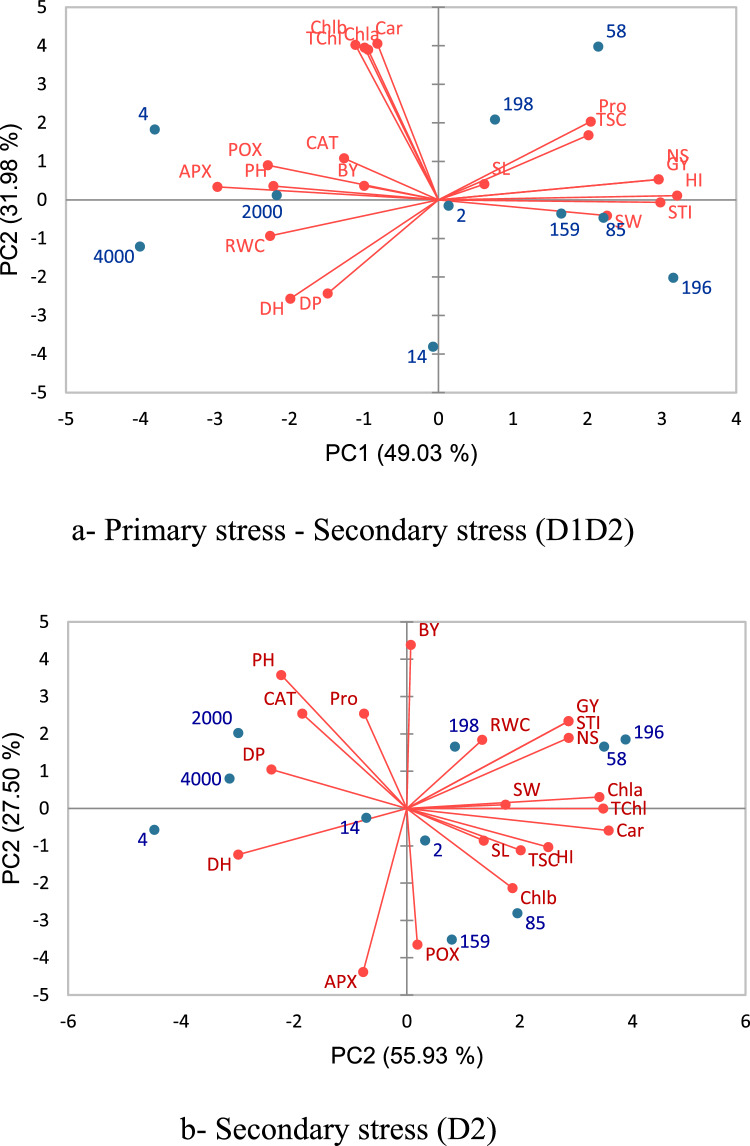
Principal component analysis of traits measured in the 10 synthetic and common wheat genotypes (**a**) under primary stress—secondary stress (D1D2) and (**b**) secondary stress (D2) conditions. Horizontal and vertical axes are the first and second principal components, respectively. DH, days to heading; DP, days to pollination; PH (cm), plant height; SL (cm), spike length; NS, spike per m2; GY (g/m2), grain yield; SW (g), thousand-grain weight; BY (g/m2), biological yield; HI (%), harvest index; STI, stress tolerance index; RWC (%), relative water content; POX (µmol/mg/protein), peroxidase; APX (µmol/mg/protein) ascorbate; CAT (µmol/mg/protein), catalase; Pro (µmol/g leaf) Proline; TSC (mg/ml) , total soluble carbohydrates; Chl-a (mg/g leaf), chlorophyll a; Chl-b (mg/g leaf), chlorophyll b; TChl (mg/g leaf), total chlorophyll a and b; Cars (mg/g leaf), carotenoids concentrations. The Number of genotypes is according to the first column (Genotype code) in table S1.

Principal component analyses (PCA) based on functional, phenological, physiological, and drought tolerance index (STI) were performed (Figs. 5 and 6). The principal component graph revealed that the first two components explained 79.20% and 81.54% of trait variation at normal condition (N) and seed priming-second stress (SD2), respectively (Fig. 5a, b). Under normal irrigation, the first principal component (PC1) had higher correlation with chlorophyll a, chlorophyll b, chlorophyll a + b, and carotenoids concentrations; the second principal component (PC2) had higher correlation with grain yield, biological yield, harvest index, number of spike (Fig. 5a).

Under seed priming-second stress (SD2), PC1 had positive correlations with total soluble carbohydrates and biological yield. PC2 positively correlated with harvest index, grain yield, number of spike and stress tolerance index (Fig. 5b) As a result, genotypes 196, 159, and 58 had high values for both PC1 and PC2 under seed priming-second stress (SD2) condition (Fig. 5b).

The first two components justified 81.01 and 83.43% of total variance at primary stress-secondary stress (D1D2) and secondary stress (D2) conditions, respectively (Fig. 6a, b). Under primary stress-secondary stress (D1D2), the first principal component (PC1) had higher correlation with stress tolerance index, harvest index, grain yield, number of spike, proline and total soluble carbohydrates (Fig. 6a). The second principal component (PC2) had high correlation with chlorophyll a, chlorophyll b, chlorophyll a + b, and carotenoids concentrations (Fig. 6a). Under secondary stress (D2) condition, PC1 had positive correlations with chlorophyll a, chlorophyll a + b, carotenoids concentrations (Fig. 6b). The second principle component (PC2) had had positive correlations with biological yield, grain yield, stress tolerance index and number of spike (Fig. 6b). As a result, genotypes 196, 58 and 199 under secondary stress condition (D2) and genotypes 159, 85 and 198 under primary stress-secondary stress (D1D2) were found to have high yield potential.

## Discussion

Plants pre-exposed to environmental stress can achieve the potential to display a stronger and faster activation of their defense system in response to the subsequent stress challenges, here defined as priming^25^. We tested whether seed priming and drought priming during the joining stage could alleviate yield reduction of intense stress imposed during anthesis in a diverse germplasm of synthetic and common wheat under field conditions.

The results of present study indicated that drought stress during the reproductive stage had considerable negative effects on yield and yield components but it was observed that drought primed plants (D1D2) reduced this negative effects compared to the non-primed plants (D2) (Table 1). Pre-exposed drought during vegetative growth proved to be a valuable strategy to facilitate wheat plants to initialize an efficient tolerance mechanism. The results confirm that drought primed plants were able to acclimate to frequent drought episodes by altering physiological factors. Indeed, plants are altering their physiology and metabolism in response to prior experience which is in agreement with other researchers in different wheat studies^1,26,27^. However, their research was restricted to a small number of cultivars and under pot conditions. The positive effects of drought priming to tolerate drought stress were more pronounced in drought primed plants (D1D2) than seed primed plant (SD2). However, seed primed genotypes (SD2) were also superior to non-primed plant (D2) in terms of biological yield and 100-seed weight. In other pervious research seed composition during terminal drought and seed priming improved the salt tolerance in wheat by modulating the water relations, osmolytes accumulation and lipid peroxidation^28^.

During water stress, physiological changes active important adaptation mechanisms for plants to resist drought^16^. Final sampling after applying reproductive stress (D2) showed that D1D2 treatment had more activated antioxidant system than the moisture treatment (D2). The development of the efficient reactive oxygen species (ROS) detoxifying system in the pre-drought stressed wheat lines indicated the existing of stress memory. The higher POX and APX activities identified in primed plants (D1D2) of this study indicated their improved redox defense status to scavenge ROS damage by down-regulating peroxidation of cell membrane lipids under water deficit stress. The effect of primary stress (D1) on physiological measured traits during recovery period was also quite evident. For example, there was a significant difference at recovery period (four weeks normal irrigation after primary stress (D1)) for some traits such as the activity of antioxidant enzymes (APX and GPX) and proline in D1D2 treatment compared to the control treatment (N). It seems that mild primary drought stress (D1) has activated the plant's physiological defense system, creating a kind of cellular readiness that will help the plant perform better than a plant which has only seen reproductive stress. We demonstrate that drought priming boosts the activity of anti-oxidative enzymes, which is vital for depressing oxidative damage and for enhancing tolerance to repeated drought stress in *T. aestivum*. These findings suggest that primary stress has led to the induction of drought stress memory mechanism and subsequently yielded improvement of genotypes under the secondary drought stress which is in agreement with Abide et al.^26^ However, our results indicated that the response of genotypes under different moisture treatments was different causing to significant effect of treatment by genotype interaction for some traits such as APX, POX and CAT. For example, the genotype number 4000 had the maximum value of POX under D1D2 treatment, while this genotype has an average value for the APX. Such responses show that stress memory is dependent on the type of genotype and this point should be considered in the breeding selection to improve this trait. The results of principal component analysis indicate a high correlation between APX and POX (The correlation coefficient between two variables is defined as the cosine of the angle between their respective vectors), which shows that the changing trend of these two variables is in the same direction and one of them can be used as a marker of the other. For developing drought tolerant varieties, selection genotypes with high yield under normal conditions and less reduction of yield under moisture stress is ideal. Some selection indices such as drought tolerance index (STI) have been developed to distinguish tolerant and non- tolerant genotypes^29^. In the present study, significant differences were observed among the 27 genotypes not only for measured traits but also for STI indicating considerable genotypic variation in the studied germplasm. Synthetic wheat genotypes had a significant superiority in terms of yield and yield components and drought tolerance (STI) compared to common wheat genotypes (local and foreign), in both normal and drought conditions. Synthetic genotypes have been obtained from the cross between tetraploid wheat (Emmer and Durum) with *Aegilops tauschii* (the wild diploid progenitor of the D genome). Genome D contains resistant genes to various biotic and abiotic stresses making it as great potential for wheat improvement. Despite little difference in antioxidant activity between synthetic and common wheat (the results of present study), large potential of synthetic wheats in terms of drought tolerance and grain production indicates that root characteristic system may play more contribution in synthetic wheat which need further studies. In a study by Song et al.^30^ synthetic wheat displayed increased plant height, larger flag leaf area, longer spikes and more biomass plant than the control cultivar. Synthetic wheat genotypes can provide new sources for grain yield potential, disease resistance, abiotic tolerance, and nutrient-use efficiency and it is becoming more and more important for modern wheat improvment^31^ to enhance yield traits to face global climate changes and feed the world’s increasing population, especially in arid environments.

Although synthetic wheat genotypes had higher grain yield in normal condition (N), seed priming-second stress (SD2), and secondary stress (D2), the difference between the three genotypic groups was not significant under primary stress-secondary stress (D1D2) (Fig. 2). Indeed, three genotypic groups had similar grain yield under primary stress-secondary stress (D1D2). It seems that drought stress memory helps common wheat genotypes to boost their performance under drought stress. The response of genotypes to stress memory was very different. Canadian wheat, which often had the lowest yield in four water conditions, had the lowest yield loss in drought primed plants (D1D2). It seems that sensitive genotypes had better response to stress memory which was in consensus with previous reports by Mendanha et al.^32^ but they had similar behavior at physiological level compare to other cultivars^27^. Therefore, the type of response to priming appears to be cultivar dependable, and thus phenotypical variation should be expected when studying the effects of abiotic priming. While three genotypic groups had specific behaviors for functional traits and secondary drought stress induced a strong synthesis of proline, carbohydrate, catalase and ascorbate peroxidase activities, there were not specific behaviors for physiological traits between three genotypic groups. In fact, among the ten genotypes that were selected in order to measure physiological traits there were not different physiological behaviors. For example, both sensitive and tolerant genotypes had higher amount of proline, catalase and peroxidase. Thus, it is very likely that differences between synthetic and common wheat genotypes, and factors that make synthetic genotypes superior to common genotypes, are more related to the genetic and cellular level than the physiological level but more studies are needed to reveal the reasons for superiority of synthetic wheats to common wheats at the physiological, biochemical and root characteristic system.

## Conclusions

It was concluded that wheat plants pre-exposed to primary moderate drought stress in a field trail retained a drought stress memory that triggered more efficient and faster stress scavenging mechanism towards severe later drought stress. Wheat lines subjected to drought priming (D1D2) showed efficient enzymatic antioxidant system leading to less reduction in grain yield. However, high genetic variation observed in term of stress memory within and between two wheat groups (synthetic and common wheat lines). Our results also indicated that the response of genotypes under different treatments was different causing to significant effect of treatment by genotype interaction for some traits such as APX, POX and CAT. Such responses show that stress memory is dependent on the type of genotype and this point should be considered in the breeding selection to improve this trait. Based on the results of PCA, genotypes number 58, 159 and 196 were recognized as the superior genotypes in term of drought tolerance. Furthermore, synthetic wheat genotypes had a significant superiority in terms of yield and yield components and drought tolerance index (STI) compared to common wheat genotypes. However, exotic cultivars, which were more sensitive to drought, exhibited better effects of stress memory in response to water deficit stress. The jointing stage primed plants (D1D2) showed higher efficient enzymatic antioxidant system and effectively alleviated grain yield inhibition than those seed primed plants (SD2). In addition to physiological studies, root characteristic system, epigenetic and molecular elucidations suggest to better understand the mechanisms of drought tolerance resulting from stress memory.

## Materials and methods

### Plant material and experimental site

Twenty-seven wheat genotypes including 20 synthetic hexaploid wheat, 4 local (Iranain) hexaploid bread wheat and 3 common Canadian hexaploid bread wheat genotypes were evaluated during 2019 and 2020. Canadian and Iranian cultivars (common wheat) were chosen among the famous commercial varieties. Synthetic lines were chosen from the collection available at the gene bank at CIMMYT (Table S1). Our plant material is a public panel and comply with relevant institutional, national, and international guidelines and legislation.

The research farm of the Isfahan University of Technology, 32–30°N, 51–20° E, Isfahan, Iran was used to conduct this study. The annual mean of temperature and precipitation were 14.4 °C and 141 mm, respectively. The soil was non-sodic and non-saline categorizing as Typic Haplargid, silty clay loam containing 389 g/kg Ca-carbonate equivalent and 49.0 g/kg organic C and 0.76 g/kg total N, with the pH of 8.2. The electrical conductivity (EC) of the farm soil-saturated extract was 1.59 dS/m and the sodium adsorption (SA) ratio was 1.39 (mmol/l).

### Treatments

The study was comprised of two separate phases. In the first phase, genotypes were evaluated at two moisture environments of well-watered and intensive drought stress (as follow) in 2018. In the second phase, seeds collected from well-watered were used to plan three priming treatments and seed collected from drought stress was used as fourth treatment. All four treatments were evaluated in 2019 and 2020 in field according to the following explanation (Fig. 1). Genotypes was sown on first November of each year. 

The experiment was conducted with four treatments: 1) normal treatment (N), plants were irrigated when 40% of the total available soil water was depleted from the root-zone. 2) Primary stress—secondary stress (D1D2) treatment, primary water stress was applied at jointing stage when 70% of the total available soil water was depleted from the root-zone for two weeks and then after applying four weeks recovery (normal irrigation), secondary water stress was applied at the anthesis stage when 90% of the total available soil water was depleted from the root-zone. 3) Secondary stress (D2), only water stress was applied at the anthesis when 90% of the total available soil water was depleted from the root-zone. 4) Seed stress—secondary stress (SD2) treatment, only water stress was applied at anthesis when 90% of the total available soil water was depleted from the root-zone and continued until physiological ripening. The seeds of fourth treatment were stressed seeds from the previous year (2018) while the seeds of other treatments were the result of normal condition of the previous year.

There were also two sampling points: firstly, at the end of the recovery period, four weeks after applying D1, samples from N and D1D2 treatments were taken. Secondly, samples were taken at the end of second stress from all treatments (N, SD2, D1D2 and D2) (Fig. 1).

Soil samples were collected every second day at depths of 0–20, 20–40 and 40–60 cm between two irrigations and exactly one day before irrigation to measure soil moisture. The irrigation depth was determined according to the following formula:

$${\text{Id}} = \, \left( {\theta_{{{\text{FC}}}} {-} \, \theta_{{{\text{irrig}}}} } \right) \, \times {\text{D}} \times \, (\rho_{{\text{b}}} / \, \rho_{{\text{w}}} )$$

where Id is irrigation depth (cm), θ_irrig_ is soil gravimetric moisture percentage at irrigating time, θ_FC_ is soil gravimetric moisture percentage at field capacity, ρ_b_ is the soil bulk density at root-zone (1.4 gcm^3^), D is the root-zone depth (60 cm) and ρ_w_ is water density. Water was delivered from a pumping station via polyethylene pipe and the water volumes for irrigation were measured with a volumetric counter.

### Measurements

A set of traits including days to heading (DH); days to pollination (DP); number of spike (NS), plant height (PH), grain yield (GY), biological yield (BY), 1000-seed weight (SW), harvest index (HI) were measured. In addition, Relative water content (RWC) was estimated according to Ritchie et al.^33^. Carotenoid, Chlorophyll a (Chla) and chlorophyll b (Chlb) were measured using Lichtenthaler and Buschmann^34^ method at the wave lengths of 661.5, 644.8 and 470 nm, respectively. The sum of Chla + Chlb was consider as total chlorophyll content (TChl). The Bates’s method^35^ was used to measure proline content. Water-soluble carbohydrate content was recorded according to Dubois et al.^36^. The activity of catalase (CAT) was determined by measuring the oxygen and water molecules generated from conversion of H_2_O_2_^37^. The activity of Ascorbate peroxidase (APX) was determined based on the oxidation of ascorbate to dehydroascorbate^38^ identified via monitoring the decrease in absorbance at 290 nm for 2 min by spectrophotometer. The method of Herzog and Fahimi^39^ was used for measuring peroxidase activity (POX) which is based on the increase in absorbance at 470 nm over the course of 2 min.

Drought tolerance index was calculated based on the seed yield under normal and drought stress conditions according to the following equation:

$${\text{STI }} = \, \left( {{\text{Ysi }} \times {\text{ Ypi}}} \right)/ \, \left( {{\text{Ymp}}} \right)^{{2}}$$

where Ymp refers to grain yield mean over all genotypes grown under normal conditions. Ypi and Ysi are seed yield of the ith genotype under normal and stress irrigation conditions, respectively.

### Statistical analysis

After normality test, three-way ANOVA indicated that year effect is non-significant for most of the traits. Therefore, the data of two years were pooled and the two-way analysis of variance (combined analysis) was performed to examine differences between the moisture environments, genotypes, and their interactions by using the general linear model (GLM) in the SAS Software32. Least significant differences (LSD) method was used for mean comparisons. Principal component analyses (PCA) were also performed based on a correlation matrix on all functional, phenological and physiological traits.

## Supplementary Information

Supplementary Information.

## Data Availability

The datasets used and/or analysed during the current study available from the corresponding author on reasonable request.
