# Supplementary material for: Drought stress memory in a germplasm of synthetic and common wheat: antioxidant system, physiological and morphological consequences
Source: Sci Rep. 2023 May 26;13:8569. doi: 10.1038/s41598-023-35642-2 (PMC10220003; doi:10.1038/s41598-023-35642-2)
Supplement: Supplementary file 1 — Supplementary Information. [file 41598_2023_35642_MOESM1_ESM.docx]

**Table S1.** Information of 27 wheat lines used in this study.

| **Genotype code** | **GID** | **INTRID** | **CID** | **SID** | **Synthetic degree** | **Pedigree** |
| --- | --- | --- | --- | --- | --- | --- |
| **Synthetic wheat** | |  |  |  |  |  |
| 7 | 2454874 | BW31682 | 72683 | 805 | 2^nd^ | Altar84/***Ae.Squarrosa*(219)**//SeriM 82 |
| 8 | 2454873 | BW31683 | 72683 | 806 | 2^nd^ | Altar84/***Ae.Squarrosa*(219)**//SeriM 82 |
| 14 | 2454969 | BW31694 | 72744 | 378 | 2^nd^ | Dverd_2/***Ae.Squarrosa*(214)**//OpataM85 |
| 17 | 2454966 | BW31697 | 72744 | 381 | 2^nd^ | Dverd_2/***Ae.Squarrosa*(214)**//OpataM85 |
| 27 | 1874477 | BW31788 | 167282 | 570 | 2^nd^ | Bacanora T88//Croc_1/***Ae.Squarrosa*** **(662)** |
| 54 | 1403557 | BW32114 | 101714 | 72 | 4^th^ | Croc 1/***Ae.Squarrosa*** **(205)**//Jupateco F73/Bluejay/3/Super Kauz/4/K |
| 58 | 1118888 | BW33631 | 72726 | 531 | 2^nd^ | Croc 1/***Ae.Squarrosa*** **(224)**//Opata M85 |
| 65 | 3616956 | BW33667 | 158452 | 132 | 3^rd^ | Filin/Irena/5/Cando/R143//Ente/Mexicali 2/3/***Aegilops Squarrosa*** **(*Taus*)**/4/Weaver |
| 80 | 3621025 | BW33746 | 167276 | 215 | 2^nd^ | Bacanora T88//Cerceta/***Ae.Searsii*** **(34D)** |
| 82 | 3616097 | BW33757 | 152383 | 95 | 3^rd^ | Altar 84/***Ae.Squarrosa* (224)**/Cucurpe S86/3/PI 610755 |
| 85 | 3584454 | BW33952 | 135084 | 149 | 3^rd^ | Croc 1/***Ae.Squarrosa*** **(205)**//Borlaug M95/3/2*Milan |
| 102 | 3855902 | BW36555 | 280805 | 59 | 4^th^ | Milan/Kauz/5/Cando/R143//Ente/Mexicali_2/3/***Aegilops Squarrosa*** **(*Taus*)**/4/Weaver/6/Tobari F66/Era//Tobari F66/Ciano F67/3/Pollo/4/Veery#5/5/Kauz |
| 135 | 3888349 | BW36956 | 334948 | 264 | 3^rd^ | Pastor/3/Altar 84/***Aegilops Squarrosa*** **(*Taus*)**//Opata M85 |
| 154 | 3832784 | BW37608 | 58520 | 92 | 2^nd^ | Chen/***Ae.Squarrosa***//2*Opata M85 |
| 159 | 4577785 | BW37701 | 342438 | 56 | 3^rd^ | SuperKauz/Pastor/3/Croc_1/***Ae. Squarrosa*(224)**//Opata M85 |
| 173 | 4883007 | BW39383 | 363192 | 46 | 3^rd^ | Croc 1/***Ae.Squarrosa*** **(224)**//Opata M85/3/Altar 84/ ***Aegilops squarrosa (Taus)***//Opata M85/4/Pastor |
| 196 | 4886016 | BW39489 | 378807 | 61 | 2^nd^ | Decoy 1/***Ae.Squarrosa*** **(458)**/3/Kauz/Gygis/Kauz |
| 198 | 4886014 | BW39491 | 378807 | 67 | 2^nd^ | Decoy 1/***Ae.Squarrosa*** **(458)**/3/Kauz/Gygis/Kauz |
| 199 | 6174895 | BW49397 | 520259 | 21 | 4^th^ | Altar 84/***Ae. Squarrosa*** **(221)**//3*Borlaug M95/3/Ures T81/Junco//Kauz/4/Weebilli/5/Mutus |
| 200 | 6174901 | BW49399 | 520259 | 27 | 4^th^ | Altar 84/***Ae. Squarrosa*** **(221)**//3*Borlaug M95/3/Ures T81/Junco//Kauz/4/Weebilli/5/Mutus |
| **Common wheat** | | **Variety** | |  |  |  |
| 4000 |  | Pishtaz | - | - | - |  |
| 2000 |  | Roshan | - | - | - |  |
| 3000 |  | Kavir | - | - | - |  |
| 1000 |  | Ghods | - | - | - |  |
| **Canadian wheat** | | **Variety** | |  |  |  |
| 1 |  | AAC Scotia | - | - | - |  |
| 2 |  | Carberry | - | - | - |  |
| 4 |  | Hoffman | - | - | - |  |

| **Table S2.** Analysis of variation for functional and phenological traits in 27 of synthetic and common wheat genotypes under two moisture environments | | | | | | | | | | |
| --- | --- | --- | --- | --- | --- | --- | --- | --- | --- | --- |
| Days to pollination | Days to heading | Height | Spike length | Harvest index | Number of spikes | Thousand grain weight | Biological yield | Grain yield | DF | **Source of**  **Variation** |
| 4952.22^**^ | 174.18^**^ | 60.78^n.s^ | 0.86^n.s^ | 6775.9^**^ | 10374536^**^ | 12904.1^**^ | 45393351^**^ | 15231673.2^**^ | 3 | Treatment |
| 4.24^n.s^ | 35.62^n.s^ | 307.73^n.s^ | 0.62^n.s^ | 341.5^n.s^ | 2706.2^n.s^ | 77.23^n.s^ | 289279^n.s^ | 157136.8^n.s^ | 12 | Rep (Treatment ) |
| 70.91^**^ | 1957.59^**^ | 466.10^**^ | 5.85^**^ | 281.7^**^ | 172876.7^**^ | 151.23^**^ | 5005015^**^ | 281243.1^**^ | 26 | Genotype |
| 3.57^n.s^ | 73.01^n.s^ | 116.72^n.s^ | 0.42^n.s^ | 62.8^n.s^ | 42328.3^n.s^ | 26.76^n.s^ | 319162^n.s^ | 34944.1^n.s^ | 78 | Treatment * Genotype |
| 4.25 | 3.21 | 119.56 | 0.64 | 88.21 | 46636.8 | 24.28 | 315322 | 54952.19 | 205 | Error |
| *: Significant at the 0.05 probability level, **: Significant at the 0.01 probability level, ns: non-significant. | | | | | | | | | | |

| **Table S3.** Analysis of variation for physiological traits in synthetic and common wheat genotypes at recovery period after primary stress | | | | | | | | | | |
| --- | --- | --- | --- | --- | --- | --- | --- | --- | --- | --- |
| POX | APX | CAT | TSC | Proline | Cars | Chl-  a+b | Chl-b | Chl-a | DF | **Source of**  **Variation** |
| 8.21^n.s^ | 2874.64^**^ | 0.620^n.s^ | 1545989.29^**^ | 54.41^**^ | 0.027^n.s^ | 0.842^**^ | 0.002^n.s^ | 0.756^**^ | 3 | Treatment |
| 54.21 | 11.88 | 0.125 | 28952.01 | 0.494 | 0.001 | 0.003 | 0.001 | 0.003 | 12 | Rep (Treatment) |
| 162.92^**^ | 325.54^**^ | 1.43^**^ | 185624.91^**^ | 6.22^**^ | 0.025^n.s^ | 0.385^n.s^ | 0.058^**^ | 0.229^n.s^ | 26 | Genotype |
| 30.24^n.s^ | 156.07^**^ | 0.648^*^ | 425631.49^**^ | 4.22^**^ | 0.014^n.s^ | 0.345^n.s^ | 0.006^n.s^ | 0.314^n.s^ | 78 | Treatment * Genotype |
| 22.52 | 17.70 | 0.212 | 37742.61 | 0.844 | 0.012 | 0.243 | 0.012 | 0.151 | 205 | Error |
| Notes. chlorophyll a (Chl-a), chlorophyll b (Chl-b) and carotenoids (Cars) concentrations, total soluble carbohydrates (TSC), catalase (CAT), ascorbate peroxidase (APX), peroxidase (POX) activities *: Significant at the 0.05 probability level, **: Significant at the 0.01 probability level, ns: non-significant. | | | | | | | | | | |

| **Table S4.** Analysis of variation for physiological traits of synthetic and common wheat genotypes in two years at the end of secondary drought stress | | | | | | | | | | | |
| --- | --- | --- | --- | --- | --- | --- | --- | --- | --- | --- | --- |
| **Source of**  **Variation** | DF | Chl-a | Chl-b | Chl-a+b | Cars | Proline | TSC | CAT | APX | POX | RWC |
| Treatment | 3 | 3.60^**^ | 0.052^**^ | 1.41^**^ | 0.289^**^ | 476.59^**^ | 9660191^*^ | 1.65 ^n.s^ | 170.19^**^ | 156.87^**^ | 130.52^n.s^ |
| Rep (Treatment ) | 12 | 0.12 | 0.004 | 0.05 | 0.011 | 3.56 | 122325 | 0.30 | 10.02 | 2.82 | 28.41 |
| Genotype | 9 | 0.27^n.s^ | 0.021^n.s^ | 0.22^n.s^ | 0.011 ^n.s^ | 9.32^**^ | 170128^**^ | 2.52^**^ | 12.55^**^ | 61.92^**^ | 165.28^**^ |
| Treatment * Genotype | 27 | 0.13^n.s^ | 0.006^n.s^ | 0.27^n.s^ | 0.007^n.s^ | 16.03^**^ | 70124^n.s^ | 2.32^**^ | 6.82^**^ | 25.96^**^ | 63.42^n.s^ |
| Error | 69 | 0.12 | 0.01 | 0.19 | 0.01 | 3.49 | 43907 | 0.34 | 2.11 | 3.92 | 35.52 |
| Notes. chlorophyll a (Chl-a), chlorophyll b (Chl-b) and carotenoids (Cars) concentrations, catalase (CAT), total soluble carbohydrates (TSC), ascorbate peroxidase (APX), peroxidase (POX) activities, Relative water content (RWC).  *: Significant at the 0.05 probability level, **: Significant at the 0.01 probability level, ns: non-significant. | | | | | | | | | | | |

|  |  |  |  |  |  |  |  |  |
| --- | --- | --- | --- | --- | --- | --- | --- | --- |
| **Table S5.** Mean comparison of harvest index and number of spikes in 27 genotypes of wheat synthetic and common | | | | | | | | |
| Number of spikes | | | | Harvest index | | | | |
| D_2_ | D_1_D_2_ | S | N | D_2_ | D_1_D_2_ | S | N | Genotype codes |
|  |  |  |  |  |  |  |  | Synthetic wheat |
| 1062.9 | 1453.6 | 1448.2 | 2601.8 | 25.82 | 37.84 | 34.75 | 44.34 | 200 |
| 1371.1 | 1760.7 | 1371.4 | 2519.6 | 31.51 | 37.54 | 36.20 | 44.31 | 80 |
| 1291.4 | 1663.4 | 1185.7 | 2437.5 | 29.42 | 36.41 | 38.42 | 43.46 | 196 |
| 1085.7 | 1233.9 | 1132.1 | 2369.6 | 34.79 | 35.65 | 32.06 | 43.54 | 159 |
| 1197.1 | 1425 | 1194.6 | 2285.7 | 27.08 | 33.30 | 31.86 | 42.93 | 8 |
| 1415.7 | 1382.1 | 1342.9 | 2260.7 | 31.47 | 36.14 | 31.96 | 36.77 | 58 |
| 1174.3 | 1201.8 | 1121.4 | 2201.8 | 25.95 | 31.09 | 30.84 | 41.19 | 154 |
| 1188.6 | 1130.4 | 1432.1 | 2160.7 | 26.41 | 33.71 | 33.4 | 39.01 | 7 |
| 951.4 | 1444.6 | 1235.7 | 2132.9 | 27.39 | 34.43 | 30.93 | 43.45 | 199 |
| 1140 | 1816.1 | 1451.8 | 2123.2 | 30.08 | 40.56 | 33.4 | 44.27 | 54 |
| 1294.3 | 1223.2 | 1371.1 | 2025 | 27.48 | 34.20 | 29.68 | 36.53 | 82 |
| 1124.3 | 1219.6 | 1053.6 | 2021.4 | 25.70 | 30.56 | 29.59 | 38.44 | 17 |
| 1127.1 | 1023.2 | 1183.9 | 1921.4 | 26.53 | 27.71 | 34.70 | 38.55 | 173 |
| 972.9 | 1151.8 | 910.7 | 1853.6 | 23.42 | 29.26 | 23.15 | 35.67 | 65 |
| 1072.9 | 1008.9 | 1291.1 | 1846.4 | 30.05 | 32.37 | 29.53 | 35.42 | 14 |
| 1190 | 1366.1 | 960.7 | 1832.1 | 27.12 | 36.36 | 30.46 | 36.74 | 198 |
| 1032.9 | 1437.5 | 1208.9 | 1764.3 | 23.44 | 34.70 | 29.74 | 41.24 | 102 |
| 927.1 | 1353.6 | 1105.4 | 1683.9 | 22.53 | 48.51 | 31.11 | 41.70 | 85 |
| 744.3 | 1235.7 | 820.5 | 1626.8 | 23.24 | 32.54 | 26.37 | 39.76 | 27 |
| 960.7 | 1366.1 | 1014.3 | 1460.7 | 24.30 | 29.43 | 26.64 | 28.56 | 135 |
|  |  |  |  |  |  |  |  | Local wheat |
| 958.6 | 1090.2 | 1119.6 | 1983.9 | 19.27 | 28.21 | 23.20 | 39.56 | 1000 |
| 994.3 | 1205.4 | 1278.6 | 2108 | 19.24 | 26.49 | 23.65 | 32.43 | 2000 |
| 734.3 | 1003.6 | 776.8 | 1958 | 18.20 | 26.55 | 23.66 | 36.45 | 3000 |
| 878.6 | 1096.4 | 680.4 | 1805.4 | 20 | 25.02 | 20.36 | 38.71 | 4000 |
|  |  |  |  |  |  |  |  | Canadian wheat |
| 721.4 | 9991.1 | 696.4 | 1175.9 | 21.35 | 26.39 | 23.48 | 26.67 | 4 |
| 977.9 | 889.3 | 876.8 | 1550 | 21.04 | 28.16 | 26.41 | 32.62 | 1 |
| 781.4 | 1414 | 1116.1 | 1753.6 | 22.85 | 34.51 | 28.29 | 35.17 | 2 |
| 369.4 | 400.9 | 513.4 | 627.4 | 5.75 | 9.53 | 7.04 | 6.42 | LSD |
| Notes. Normal condition (N), seed priming-secondary stress (SD2), primary stress-secondary  stress (D1D2) and secondary stress (D2)**.** Mean followed by the same letter in each column  are not significantly different according to LSD test (probability level of 5%). | | | | | | | | |
